# Viral suppression among young adults in a US outpatient clinic

**DOI:** 10.1186/1471-2334-14-S2-P76

**Published:** 2014-05-23

**Authors:** Enbal Shacham, Amy Estlund, Rachel Presti

**Affiliations:** 1 Saint Louis University, Saint Louis, USA

## Introduction

HIV infection has become a chronic disease for populations with access to medical care and treatment. Regardless of medical advancements, new infections persist. With young adults being those that are most often newly infected, research needs to be conducted to assess medication adherence barriers, specific to young adults with HIV.

## Materials and methods

As standard of care, patients presenting for care completed behavioral and psychological distress assessments annually. Data were abstracted from medical charts to include both the self-reported data and HIV parameters in 2013 among patients aged 18 and 30 years. Descriptive and regression analyses were conducted to identify factors related to viral suppression (< 20 copies/mL).

## Results

A total of 335 individuals presented for care during the 12 month period. The majority were African American (84%, n=281), and had a mean age of 25.1 (SD = 2.8). Nearly all (n = 305; 91%) had current prescriptions antiretroviral therapy (ART); among those receiving ARTs, 72.8% were virally suppressed. Sexually transmitted infection tests are conducted annually and by assessed need; 30% of this sample had at least 1 STI diagnosis (gonorrhea, chlamydia, syphilis, trichomoniasis) within the last year. Nearly 60% reported condom use at last sex, which was not associated with viral load suppression. Women more often had unsuppressed viral loads (p< 0.001). Significant proportions of the sample expressed moderate to severe depressive and anxiety-type symptoms; which was more common among individuals who were not virally suppressed (n = 230; p< 0.001). Additionally, 13% endorsed suicidal ideation within the past 2 weeks; which was also associated with viral suppression (p< 0.001). Further, 3% reported injecting drugs, this suggests new trends in HIV infection regionally.

## Conclusions

These findings related to how young adults are managing their HIV care suggest increased efforts to preventing additional STIs, managing psychological distress, and improving opportunities for female patients. Applying lessons from global settings to U.S. settings may provide great insight to improving engagement in HIV care, as has been done successfully in many international settings.

